# A Retrospective Cohort Study of Women and Men Living with HIV, Attending an HIV Clinic in Australia

**DOI:** 10.1089/whr.2022.0038

**Published:** 2022-11-09

**Authors:** Rochelle A. Hamilton, Yvonne Wells, Peter Higgs

**Affiliations:** ^1^ School of Nursing and Midwifery, La Trobe University, Bundoora, Australia.; ^2^ Infectious Disease Unit, University Hospital Geelong, Barwon Health, Geelong, Australia.; ^3^ Australian Institute for Primary Care & Ageing, La Trobe University, Bundoora, Australia.; ^4^ Department of Public Health, La Trobe University, Bundoora, Australia.; ^5^ Behaviours and Health Risks, Burnet Institute, Melbourne, Australia.

**Keywords:** gender differences, HIV, sexual health, sexual risk, women's health

## Abstract

**Objectives:**

To compare women with men presenting with HIV to a public health HIV clinic, to identify the special characteristics and health care needs of women living with HIV in the Barwon South West region in Victoria.

**Methods:**

A retrospective cohort study of 35 women and 135 men living with HIV who attended the clinic between 2009 and 2020. Gender differences were assessed using nonparametric analyses.

**Results:**

The women were diagnosed with HIV younger than the men (mean 29.5 years vs. 36.7 years) and more were born in Africa (28.6% vs. 5.2%). More men than women presented with sexually transmittable infections (38.5% vs. 14.3%) at the time of diagnosis, and were diagnosed through a sexual health screen (37% vs. 17%). The proportions of men and women who used alcohol and other drugs (recent to their diagnosis) were similar (68.1% vs. 48.6%), and there was no difference in proportions presenting with AIDS-defining illnesses (*p* = 0.425), or CD4 cell count (advanced: ≤200 cells, relatively well: ≥201 cells, *p* = 0.241), but the women had a lower viral load (*p* < 0.001).

**Conclusions:**

In this study of 170 people living with HIV, nearly one-half of the men with known HIV history were diagnosed through sexual health screens, but women's HIV was mostly detected through targeted screening. Results highlight gender disparity in access to sexual health screening and assessment, including low awareness of sexual health risks for women, and endorse the view that HIV is a heterosexual sexually transmittable infection in women.

## Introduction

Half of the global population of people living with HIV are women^1^ but they remain underrepresented in research.^2^ The HIV Futures 9 Survey report^3^ highlighted that women and men living with HIV have very different experiences. The disparities primarily concern service distribution and that many women living with HIV also carry a carer role^4^ and may experience more stigma than men do when they acquire an HIV diagnosis, which can affect their social relationships and moral identity.^5–7^ Women are at higher risk of acquiring HIV than is often acknowledged, as crucial factors in transmission risk include a complex mix of biological susceptibilities and socioeconomic and epidemiological factors.^5,8^

The biological factor is due to both the greater surface area of mucosa in a woman's genital region than in men's and a high volume and concentration of viral load (VL) in seminal fluid, making women vulnerable to the transmission risk of HIV during penile–vaginal sexual intercourse.^5,9^

Sociological risk factors that apply particularly to women include low levels of education, un- or underemployment, and insecure housing, which render them more likely to depend on their partner's family for housing and financial support. Women also face higher risk of sexual abuse and partner infidelity than men.^9–13^

About 3,000 women currently live with HIV in Australia,^14^ representing ∼10% of the total population of Australians diagnosed with HIV.^4^ It was estimated in 2020 that between 400 and 500 women living with HIV were unaware of their status.^15^ In Victoria, between 1994 and 2016, there were 465 newly diagnosed women living with HIV.^4^ Of those, the majority were aged between 20 and 39 years, with 16% stating they lived in regional or remote Victoria.^4^ From 2015 to 2019, women accounted for 11.3% of newly acquired HIV nationally.^14,16^

In the Barwon South Western (BSW) region of Victoria, women comprise a higher proportion of the population living with HIV than the national average.^17,18^ Nationally, women account for ∼13.7% of the population living with HIV,^19^ and ∼16% of those living with HIV in Victoria.^4^ In the BSW region, in contrast, women living with HIV account for between 23% and 40% of the total HIV cohort presenting to the HIV clinic (unpublished hospital attendance data). Generally, in Australia the burden of disease and mortality rate increases with increasing geographical distance.^20–23^ For example, compared with people living in major cities, screening rates for bowel, breast, and cervical cancer are much lower, resulting in higher rates of potentially preventable hospitalization.^24^ Most health services outside metropolitan regions do not cater for specialist care, such as support for high-risk pregnancies or for people with blood-borne viruses (BBVs).

Together, these access barriers place women living with HIV in regional and rural areas in a vulnerable position. Furthermore, their unmet care needs are likely to be very different from those of women residing in metropolitan settings^25^ because of the absence of connections—family, access to peer-based programs, and extended community—and a higher real and perceived risk around privacy and confidentiality.^3,17,18^

Australian data on gender differences in the characteristics of people living with HIV and residing in regional areas have not previously been explored. This study aimed to describe the characteristics of people with HIV attending the BSW HIV clinic and to compare female with male patients as a first step in understanding how their needs and experiences may differ.

## Methodology

The study was approved by two Human Research Ethics Committees: Barwon Health Geelong and La Trobe University Melbourne (HREC/66370/VICBH-2020-223442; HREC/64279/VICBH-2020-217361).

### Study setting and cases included

The clinic is unique in both location and function, situated in Geelong Victoria—1 hour's drive southwest of Melbourne (75 km)—in an outpatient setting within the tertiary public hospital. The clinic aims to deliver education; prevention and management of HIV and sexually transmitted infections (STIs); and contraceptive care. While Geelong is classified as a city—the largest regional city in Victoria^26^—its catchment includes the BSW region of Victoria, which has a population of ∼430,000^27^ and spans an area of ∼1,250 km^2^.^27^ The clinic also sees patients up to and across the South Australian border, requiring some patients to travel up to 350 km each way to attend appointments. The clinic has always relied on telehealth to support many patients and their general practitioners (GPs).

A retrospective examination of medical records was completed for 35 women and 135 men who presented at a single HIV and sexual health clinic between January 1, 2009, and June 30, 2020, and were attending as new patients or already living with HIV and in the clinic's care. All data included in the study pertained to when patients were diagnosed or first presented to the clinic. Despite every effort being made to access all relevant health information at the time of diagnosis or upon entering the clinic's care, this proved unachievable. Factors impacting the ability to obtain all the data included transfer from interstate or inability to access the person's diagnostic pathology report.

### Measures

All measures were derived from data available in (most) case records and included the following:

1. Sociodemographic indicators: gender, age, sexual identity, occupational status, country of birth (CoB), and postcode.
2. HIV history, including how the illness was detected, CD4 cell count, HIV VL, and year antiretroviral therapy (ART) commenced.
3. Physical and mental health indicators: alcohol and drug use, mental health diagnoses, AIDS-defining illnesses, and other illnesses.

Because of small numbers and poor or inconsistent recording of crucial patient characteristics, several measures were derived by pooling categories to enable valid analysis, as outlined below. Where possible, pooling occurred using meaningful groupings, but some recodes were *post hoc* and depended on the numbers represented in subgroups of patients.

*Sexual identity:* How the participant self-identified was documented directly into the clinical notes. The categories nominated included men who have sex with men (MSM), bisexual, and heterosexual. MSM and bisexual categories were grouped for analysis.
*Country of birth:* The WHO defines six geographical regions.^28^ In this study, countries were grouped into Australia, Africa, and other/missing.
*Employment status:* Categories were employed, pension, and unemployed/student.
*Socioeconomic status:* A Socioeconomic Index for Areas (SEIFA)^29^ score was identified from each patient's postcode of residence. The Australian Bureau of Statistics (ABS) calculates SEIFA indices from education, occupation, and employment figures. The lower the number, the lower the socioeconomic status of the area. SEIFA deciles were grouped into three categories: highest = 8–10, middle = 5–7, and lowest = 1–4.
*Locality:* Postcode was also used to code regions as indicated using the Modified Monash Model (MMM) classifications table (metropolitan, regional centers, and rural towns).^22,23^
*How detected:* Detection of HIV status was grouped into three types. Category 1: Presented unwell, refers to being diagnosed secondary to an unrelated event and included unexpected findings and presented unwell. Category 2: Routine sexual health screen, refers to detection through a routine sexual health screen, usually undertaken on patient request. Category 3: Targeted risk, refers to other situations where tests were undertaken because there was a perception of risk. This included notification by a sexual partner or partner notification officer, immigration screening, mother-to-child-transmission, and antenatal screening, and was usually undertaken at the behest of a health professional. Category 4 comprised unknown or missing information.
*Alcohol and other drug use (AoD):* Some indicators (*e.g.*, excessive use of alcohol [past or current], and use of cigarettes) were sufficiently clear in the medical records to analyze without further grouping, noting the National Health and Medical Research harmful alcohol guidelines (NHMRC).^30^ Drugs grouped as illicit drugs included benzodiazepines, methamphetamines, ecstasy, gamma-hydroxybutyrate, intravenous drug use not specified, polysubstance use not specified, cannabis, amphetamines, steroids, OxyContin, heroin, cocaine, and opioids.
*Mental health diagnoses:* Patients' diagnoses at intake were recorded consistent with the *Diagnostic and Statistical Manual of Mental Disorders*, 5th edition.^31^ Measures included diagnosis of an affective disorder (depression and anxiety). A summary measure of any mental health diagnosis was also constructed.
*AIDS-defining illnesses:* The WHO^32^ classification of AIDS-defining illness includes pneumocystis jirovecii pneumonia; esophageal candidiasis; Kaposi's sarcoma; chronic herpes simplex infection or herpetic esophagitis; cryptococcosis; cryptosporidiosis; toxoplasmosis; cytomegalovirus infection; mycobacteriosis, including tuberculosis; lymphoma; HIV encephalopathy; HIV wasting disease; recurrent bacterial pneumonia; and progressive multifocal leukoencephalopathy. Diagnoses were grouped to form a summary measure of any AIDS-defining illness.
*Sexually transmitted infections:* All STIs were grouped to form a summary measure including chlamydia, gonorrhea, syphilis, hepatitis B, hepatitis C, bacterial vaginosis, and human papillomavirus.
A summary measure of other medical illnesses present at the time of diagnosis included chronic liver disease, diabetes, hyperlipidemia, and Crohn's disease.
*CD4 T lymphocyte (CD4) cell counts*: Results were divided according to the susceptibility of acquisition to opportunistic infections and staging of late diagnosis.^32,33^ Two criteria were used. Criterion 1 compared “advanced” (CD4 ≤ 200 cell/mm^3^) and “relatively well” (CD4 ≥ 201 cell/mm^3^) patients, while Criterion 2 compared “late” (CD4 ≤ 350 cell/mm^3^) and “relatively timely” (CD4 ≥ 351 cell/mm^3^) patients.^33,34^
*HIV VL:* Risk of transmission of HIV was grouped into “extremely low risk of transmission” (VL ≤200 copies) or “risk of transmission” (VL = ≥ 201 copies).^33^
*Antiretroviral therapy:* The year ART was commenced, as documented in hospital records.

### Analytic strategy

Analyses were performed using SPSS v27^35^ and explored gender differences in measures. Given the categorical nature of most measures, the preferred statistic was *χ*^2^. Where men's and women's records differed on levels of missing data, analyses were undertaken not including this category. Gender differences in age at presentation were explored using a nonparametric statistic (standardized Mann–Whitney *U*). A *p*-value of 0.05 was selected to indicate statistical significance.

## Results

### Sociodemographic characteristics

Gender was documented as male (*n* = 135) or female (*n* = 35). Other possible categories (*e.g.*, transgender, nonbinary, or other) were not documented in the patient records examined. While most women identified as heterosexual, with one woman identifying as bisexual, most of the men (69%) identified as MSM, 17% as heterosexual, and 6% as bisexual. Sexual identity was undocumented for 8% of the men.

Employment status did not differ by gender, with almost one-half of both men and women reporting they were employed (see Table 1).

**Table 1.** Demographic Characteristics of Patients Diagnosed with HIV

	Women (*n* = 35)		Men (*n* = 135)		Standard U	*p*
	Mean (SD)		Mean (SD)			
	29.5 (12.1)		36.7 (14.2)		2.62	0.009
Age at diagnosis	*n*	%	*n*	%	χ^2^	*p*
CoB					16.94	<0.001
Australia	20	57.1	105	77.8		
Africa	10	28.6	7	5.2		
Other/missing	5	14.3	23	17.0		
Employment status					2.96	0.228
Employed	12	34.3	56	41.5		
Pension	16	45.7	66	48.9		
Other/missing	7	20.0	13	9.6		
Social economic status^a^					1.87	0.393
Top (SEIFA deciles 8–10)	9	25.7	40	29.6		
Middle (SEIFA deciles 5–7)	5	14.3	30	22.2		
Bottom (SEIFA deciles 1–4)	21	60.0	64	47.4		
Missing	0	0	1	0.7		
MMM zones^a^					2.14	0.343
Metropolitan	26	74.3	83	61.5		
Regional centers	3	8.6	24	17.8		
Rural locations	6	17.1	24	17.8		
Missing/interstate	0	0.0	4	3.0		
Sexual identity					75.4	<0.001
Heterosexual	33	94.3	23	17.0		
Other (MSM, bisexual)	1	2.9	101	74.8		
Missing	1	2.9	11	8.1		

^a^ Analysis omits missing values.

CoB, country of birth; MMM, Modified Monash Model; MSM, men who have sex with men; SD, standard deviation; SEIFA, Socioeconomic Index for Areas.

Age at diagnosis ranged from 12 to 79 years. Women were diagnosed with HIV at a younger age than men—mean of 29.5 years compared with 36.7 years for the men (*p* = 0.009).

The men and women in this study came from 24 countries and all 6 WHO regions.^36^ Of the 35 women, 20 were born in Australia: the remaining 15 women identified 11 other countries of birth. Australia was identified as CoB for 105 of the men; the remaining 30 men identified 16 other countries. Too few patients identified as Aboriginal or Torres Strait Islander to be able to include this category in descriptive analyses.

The majority of men and women resided in zones 1 and 2—metropolitan and regional centers.^22,23^ Forty-nine men and nine women resided outside of these zones, with 17.1% of the women and 19.4% of the men residing in zones 3, 4, and 5 (large, medium, and small rural towns).^22,23^ However, 11.4% of the women and 8.1% of the men resided in small rural towns (zone 5). More than half of the women in this study (57.1%) resided in lower socioeconomic postcodes. The socioeconomic status associated with postcode did not differ by gender (*p* = 0.873).

### HIV history

The way in which the person was notified or informed of their HIV status was generally well documented. Most frequently, patients presented unwell to the emergency department, sexual health service, or their GP (see Table 2).

**Table 2.** HIV History

	Women (*n* = 35)		Men (*n* = 135)		χ^2^	*p*
	*n*	%	*n*	%		
How HIV was detected					51.71	<0.001
Presented unwell	12	34.3	54	40.0		
Routine sexual health screen	6	17.1	50	37.0		
Targeted screening	16	45.7	4	3.0		
Unknown	1	2.9	27	20.0		
CD4 group: Criterion 1^a^					2.21	0.137
Advanced: ≤200 cells	7	20.0	26	18.3		
Relatively well: ≥201 cells	24	68.6	43	31.9		
Unknown	4	11.4	66	48.9		
CD4 group: Criterion 2^a^					5.66	0.017
Late: ≤350 cells	10	28.6	40	29.6		
Relatively timely: ≥351 cells	21	60.0	29	21.5		
Unknown	4	11.4	66	48.9		
VL group^a^					30.3	0.001
≤200 Copies (low risk)	7	20.0	3	2.2		
≥201 Copies (risk)	23	65.7	53	39.3		
Unknown	5	14.3	79	58.5		
Year ARTs commenced					3.92	0.141
Up to 2014	9	25.7	57	42.2		
2015–2020	13	37.1	32	23.7		
Missing	13	37.1	46	34.1		

^a^ Analysis omits missing values.

ART, antiretroviral therapy.

Analysis of how HIV was detected indicated significant gender differences. Women more frequently experienced the result of their HIV status due to undergoing “targeted screening” initiated by a health professional, while more men reported being notified of their positive HIV status through “routine sexual health-related screening.”

VL was not documented (or accessible) for 79 men and 5 women, and CD4 cell count was not documented (or accessible) for 66 men and 4 women at the time of diagnosis or first transfer to care. There were no significant gender differences for CD4 cell count ≤200 cells (*p* = 0.137). However, where the CD4 cell count threshold was ≤350 cells, women emerged as having received timelier diagnosis than the men (*p* = 0.017) and their VL was lower (*p* = < 0.001) (see Table 2).

ART commenced for the men between 1997 and 2019, with 11 men commencing ART in 2015. For 22 of the 35 women, ART commenced between 2004 and 2019, with four commencing in 2015 (see Table 2). This difference was not statistically significant.

### Physical and mental health indicators

AoD domains, including illicit drug use (current or recent), were documented for each individual (see Table 3). Polysubstance use was identified in some histories, but in others, individual drugs were specified. Recent injecting drug use and cannabis and/or alcohol consumption—to excess—were also identified. A higher proportion of men than women were using a substance at diagnosis.

**Table 3.** Physical and Mental Health Indicators

	Women (*n* = 35)		Men (*n* = 135)		χ^2^	*p*
	*n*	%	*n*	%		
Drug or alcohol use (any)	17	48.6	92	68.1	4.63	0.047
Illicit drugs	11	31.4	51	37.8	0.48	0.487
Alcohol (overuse)	10	28.6	53	39.3	1.36	0.243
Cigarettes	11	31.4	69	51.1	4.32	0.038
Mental health (any diagnosis)	26	74.3	44	60.0	2.43	0.169
Anxiety	14	40.0	40	29.6	1.38	0.240
Depression	19	54.3	56	41.5	1.85	0.174
AIDS-defining illness at diagnosis	15	42.9	48	35.6	0.63	0.425
STIs at diagnosis	5	14.3	52	38.5	7.32	0.007
Additional medical illness at diagnosis	1	2.9	13	9.6	0.02	0.305

STI, sexually transmitted infection.

No drug or alcohol use at all, including cigarettes, was found in the records of 31.9% of men and 51.4% of women. This difference was statistically significant (*p* = 0.047). More men (51.1%) than women (31.4%) reported current or previous cigarette smoking. However, the use of illicit drugs did not differ between the men and women (*p* = 0.487).

Mental health conditions described in the database included depression, anxiety, personality disorders, schizophrenia, self-harm, panic attacks, obsessive-compulsive disorder, paranoia, post-traumatic stress disorder, (severe) insomnia, autism spectrum disorder, bipolar disorder, psychosis, and eating disorders. Some patients had more than one recognized mental health condition recorded (see Table 3).

Depression and anxiety were the most common diagnoses, with 49.6% of the men and 65.7% of the women having either one or both diagnoses. One-fifth of the men (20%) were diagnosed with depression only, and 8.1% were diagnosed with anxiety alone: 21.5% were diagnosed with both. One-quarter of the women (25.7%) were diagnosed with depression only and 11.4% were diagnosed with anxiety alone: 28.6% were diagnosed with both. Gender differences in proportions were not statistically significant (see Table 3).

Patients' histories included AIDS-defining illnesses, with 12.6% of the men and 8.6% of the women having three or more of these. There were no significant gender differences in AIDS-defining illnesses (see Table 3).

Documented STIs included chlamydia, gonorrhea, and syphilis. Almost one-quarter of the men (22.2%) and one-in-twenty of the women (5.7%) had at least one of these additional STIs. Overall, men presented with more STIs than women (*p* = 0.007) (see Table 3).

## Discussion

This unique study compared women and men living with HIV in a regional area of Victoria, as a first step in understanding the women's experience. Despite the small number of women in the study, they represented most women living with HIV in this region.

Although the men and women who presented to the Barwon Reproductive and Sexual Health clinic in the BSW region of Victoria were similar in many respects, this study highlighted the special characteristic of the women. The journey that the women had taken to acquiring HIV and attending the clinic was quite different from that experienced by the majority of clients—the men—demonstrating the need for special consideration of the women and further exploration of their experience.

### Sociodemographic characteristics

Conditions that influence the overall health status of individuals are expressed collectively as social determinants of health and include where a person is born, raised, and resides and the employment opportunities.^37^ The current study identified no significant gender differences at the time of diagnosis or initial presentation to the clinic in geographical locality or occupational or socioeconomic status. However, the women were diagnosed with HIV at a younger age, and significantly more HIV-positive women than men were born in Africa.

Research in other countries (including India, sub-Saharan Africa, east and southern Africa, eastern Europe, and central Asia) where the sociodemographic characteristics of men and women living with HIV have been compared^9,38–40^ has also shown that HIV diagnosis occurs at an earlier age in women than men. Also, consistent with our study and previous Australian findings,^4^ a considerable proportion of the women in Campbell's USA study^40^ were born in sub-Saharan Africa, although the majority identified as African American or Latino.

In contrast, previous studies have identified indicators of relative social disadvantage in women living with HIV that were not apparent in our samples. For example, Alvarez-Uria et al^39^ found that more women living with HIV than men were unemployed, and the women were usually less well-educated than their partners and depended upon their partner or relatives for housing and financial security.

Similarly, studies from South Africa, the United States, and international collaborations^9^ have identified gendered vulnerabilities in HIV acquisition and poor outcomes following diagnosis associated with culture, language barriers, and women's dependency on their male partner for financial security and housing. Bautista-Arredondo et al^10^ were so struck by gender differences in sociodemographic characteristics of people living with HIV in Mexico City that they titled their study “A tale of two epidemics,” noting that the women with HIV were characterized by social and economic vulnerability, but the men by risky sexual behaviors.

While gender differences in demographic characteristics were not as dramatic in our study, more women than men in our sample were born in countries with a poor health system infrastructure, which is likely to have a strong impact on health literacy and how a health service is viewed. Women's experience with any comorbidities—for example, mental health problems—and previous negative experiences with government-funded services may also hinder appropriate or timely engagement with health services in Australia.

### HIV history

Women's HIV was more likely than men's to have been detected through a targeted screen. Such screens are initiated by someone other than the patient and are not in the women's control. The women did not consider they were at risk of acquiring HIV (nor were they considered to be in the risk categories) beforehand. The idea that they were at risk of HIV acquisition was totally unexpected. In contrast, more than one-third of the men had participated—through their choice—in a sexual health screen that included an HIV test. The routine screening provided to the men in this study reflects good practice in HIV prevention and permits early diagnosis of HIV and early initiation of treatment and care.

Initiating a routine sexual health screen and being advised to have an HIV screen because of external circumstances outside of one's control are very different experiences. This gender difference highlights the need to explore women's experiences in their HIV detection journey.

Moreira et al^4^ reviewed and described the characteristics of Australian women living with HIV. The study recognized many barriers to HIV testing, including access to tests, cultural restrictions, and the fear some women experience regarding attending a clinic for an HIV screen and the possibility of being diagnosed with HIV. This fear is related to the risk of abandonment by their partners, family, and community^4^ Moreira et al concluded that women with HIV present late for diagnosis for these reasons.

The current study reviewed all available data that could contribute to defining late or delayed diagnosis of HIV.^32^ Data from the women alone would support Moreira's findings—as CD4 cell count, VL, and AIDS-defining illnesses collectively describe late or advanced HIV/AIDS^33^ in a high proportion of women. However, when data on these indicators were compared with those from the men, the current study suggested few significant gender differences, and these were in favor of the women. Nevertheless, it could be argued that the clinical presentations for both the women and men presenting to the HIV clinic in the BSW region indicated delayed diagnosis, given that a clinically significant proportion of both women and men had an AIDS-defining illness and a low CD4 cell count at the time of their HIV diagnosis. Both the men and the women in this study were presenting late.

### Health and mental health

The men and women in this study were similar in their use of recreational or narcotic drugs and mental health diagnoses, but their drug-use and health profile were different—fewer women were current or former smokers than the men, and fewer had an STI at the time of their HIV diagnosis.

It is possible that higher rates of STIs in the men and the higher rate of HIV detection through sexual health screens reflect both the relatively high prevalence of STIs among gay and bisexual men in Victoria^6,41,42^ and a high awareness of HIV risk in this group due to targeted health promotion and high community engagement. In comparison, women at risk of HIV infection represent a neglected group.

### Barriers to HIV testing

Women have been recognized as a priority population in Victoria's HIV strategy.^18^ However, the heightened risk of harm to women from HIV acknowledged in the report is not yet reflected in practice in primary care. Despite women having similar rates of AIDS-defining illnesses as the men in this study, for most there was no documentation of previous routine HIV testing in the context of a sexual health screen.

Primary care is the most common first point of health access at which women of all ages present. Many studies continue to demonstrate the barriers women experience when attending primary health physicians, including medical professionals' discomfort when inquiring about sexual health and sexual behaviors.^43,44^ To improve overall HIV prevention and treatment strategies, it is vitally important to increase HIV testing and ensure access to care and improved sexual health assessments (including taking a sexual history) in primary care consultations. Maximizing this opportunity for timely HIV response for women in the community is challenging but necessary.

### Strengths and limitations

The strength of this study is the comprehensiveness of the data retrieved from medical records. A limitation of this study is the low number of patient records, which precluded some subgroup analyses including stratification by sexual identity. The records of some women were not able to be included in this study due to inability to access their information following transfer to GP care or moves interstate or to metropolitan services. In addition, many of the male participants' records had relatively high levels of missing data.

## Conclusion

Despite presenting with similar health status to men at diagnosis, the women in our study were considered low risk for STIs and BBVs and were relatively rarely offered routine sexual health screening. Most of the women only became aware of their HIV status as a direct result of targeted screening in the context of explicit risk.

The implications for policy and practice include the need to improve education about the risk of HIV acquisition across primary health (GPs), the imperative to expand sexual health assessments for women overall, and the need for further research on the sexual behaviors of women, with a view to improving understanding of their risk of HIV acquisition.

## Data Availability Statement

The data are not publicly available because of potential ethical issues but this can be negotiated with the authors.

## Abbreviations Used

ART: antiretroviral therapy
AoD: alcohol and other drug use
BBV: blood-borne virus
BSW: Barwon South Western
CoB: country of birth
GP: general practitioner
MMM: Modified Monash Model
MSM: men who have sex with men
SD: standard deviation
SEIFA: Socioeconomic Index for Areas
STI: sexually transmitted infection
VL: viral load
